# Diagnostic Performance of CT FFR With a New Parameter Optimized Computational Fluid Dynamics Algorithm From the CT-FFR-CHINA Trial: Characteristic Analysis of Gray Zone Lesions and Misdiagnosed Lesions

**DOI:** 10.3389/fcvm.2022.819460

**Published:** 2022-03-22

**Authors:** Yang Gao, Na Zhao, Lei Song, Hongjie Hu, Tao Jiang, Wenqiang Chen, Feng Zhang, Kefei Dou, Chaowei Mu, Weixian Yang, Guosheng Fu, Li Xu, Dumin Li, Lijuan Fan, Yunqiang An, Yang Wang, Wei Li, Bo Xu, Bin Lu

**Affiliations:** ^1^Department of Radiology, Fuwai Hospital, National Center for Cardiovascular Diseases, Peking Union Medical College and Chinese Academy of Medical Sciences, Beijing, China; ^2^Department of Cardiology, Fuwai Hospital, National Center for Cardiovascular Diseases, Peking Union Medical College and Chinese Academy of Medical Sciences, Beijing, China; ^3^Department of Radiology, Sir Run Run Shaw Hospital, Zhejiang University School of Medicine, Hangzhou, China; ^4^Department of Radiology, Beijing Chao-Yang Hospital, Capital Medical University, Beijing, China; ^5^Department of Cardiology, Qilu Hospital, Shandong University, Jinan, China; ^6^Department of Cardiology, TEDA International Cardiovascular Hospital, Tianjin, China; ^7^Department of Cardiology, Sir Run Run Shaw Hospital, Zhejiang University School of Medicine, Hangzhou, China; ^8^Department of Cardiology, Beijing Chao-Yang Hospital, Capital Medical University, Beijing, China; ^9^Department of Radiology, Qilu Hospital, Shandong University, Jinan, China; ^10^Department of Radiology, TEDA International Cardiovascular Hospital, Tianjin, China; ^11^Medical Research and Biometrics Center, Fuwai Hospital, National Center for Cardiovascular Diseases, Peking Union Medical College and Chinese Academy of Medical Sciences, Beijing, China

**Keywords:** CT-FFR, CT angiography, computational fluid dynamics, gray zone, diagnostic performance

## Abstract

To assess the diagnostic performance of fractional flow reserve (FFR) derived from coronary computed tomography angiography (CTA) (CT-FFR) obtained by a new computational fluid dynamics (CFD) algorithm to detect ischemia, using FFR as a reference, and analyze the characteristics of “gray zone” and misdiagnosed lesions. This prospective multicenter clinical trial (NCT03692936, https://clinicaltrials.gov/) analyzed 317 patients with coronary stenosis between 30 and 90% in 366 vessels from five centers undergoing CTA and FFR between November 2018 and March 2020. CT-FFR were obtained from a CFD algorithm (Heartcentury Co., Ltd., Beijing, China). Diagnostic performance of CT-FFR and CTA in detecting ischemia was assessed. Coronary atherosclerosis characteristics of gray zone and misdiagnosed lesions were analyzed. Per-vessel sensitivity, specificity and accuracy for CT-FFR and CTA were 89.9, 87.8, 88.8% and 89.3, 35.5, 60.4%, respectively. Accuracy of CT-FFR was 80.0% in gray zone lesions. In gray zone lesions, lumen area and diameter were significantly larger than lesions with FFR < 0.76 (both *p* < 0.001), lesion length, non-calcified and calcified plaque volume were all significantly higher than non-ischemic lesions (all *p* < 0.05). In gray zone lesions, Agatston score (OR = 1.009, *p* = 0.044) was the risk factor of false negative results of CT-FFR. In non-ischemia lesions, coronary stenosis >50% (OR = 2.684, *p* = 0.03) was the risk factor of false positive results. Lumen area (OR = 0.567, *p* = 0.02) and diameter (OR = 0.296, *p* = 0.03) had a significant negative effect on the risk of false positive results of CT-FFR. In conclusion, CT-FFR based on the new parameter-optimized CFD model provides better diagnostic performance for lesion-specific ischemia than CTA. For gray zone lesions, stenosis degree was less than those with FFR < 0.76, and plaque load was heavier than non-ischemic lesions.

## Introduction

Invasive fractional flow reserve (FFR) is the current reference standard for determining the hemodynamic significance of coronary artery disease (CAD) (1–3). FFR has been shown to be a powerful tool to identify patients with CAD who may benefit from revascularization and reduced the rate of composite endpoints (4–8). Coronary computed tomography angiography (CTA) has emerged as a non-invasive modality for visualizing coronary anatomy and plaque characteristics, and has been shown to have high diagnostic accuracy to detect and exclude obstructive CAD (9–11). FFR derived from coronary CTA (CT-FFR) using computational fluid dynamics (CFD) has been proposed and validated in prospective clinical trials as a non-invasive technique to improve the determination of ischemia lesions over CTA alone (12–15). FFR has a “gray zone” of ischemic threshold value (between 0.75 and 0.80) (2). When evaluating values close to the threshold, lower agreement has observed for repeated measures of FFR (16). Therefore, the accuracy of CT-FFR in this area is still expected to be improved. Previous studies found that CT-FFR around the cut-point of 0.80 showed less certainty, and that CT-FFR values of >0.90 and ≤0.60 provided almost complete certainty (17). However, further analysis of these lesions is limited.

The computation of CT-FFR requires multiple factors including hemodynamic parameters specified in the boundary conditions of CFD models, which cannot be directly derived from CTA. The accuracy of CT-FFR improves when those parameters are optimized for the target population. Therefore, a new parameter-optimized CFD algorithm was developed to minimize the difference between CT-FFR and FFR, using reduced-order models on retrospectively collected patient data. The diagnostic performance of the algorithm in clinical settings has not been evaluated against various real-world conditions, such as image quality and total plaque burden. In addition, the accuracy of CT-FFR can be continuously improved by analyzing of the characteristics of misdiagnosed lesions and “gray zone” lesions with caution.

The purpose of this clinical trial conducted at multiple centers in China was to assess the diagnostic performance of CT-FFR with a new parameter-optimized CFD algorithm to detect ischemia using FFR as a reference, and further analysis of coronary atherosclerosis characteristics of “gray zone” lesions and misdiagnosed lesions.

## Materials and Methods

### Study Design

The CT-FFR-CHINA (Computed Tomography Derived Fractional Flow Reserve for Coronary Hemodynamic Ischemia Non-invasive Assessment) is a prospective, multicenter trial (NCT03692936, https://clinicaltrials.gov/) designed to evaluate the diagnostic performance of CT-FFR calculated on a parameter-optimized CFD algorithm in identifying ischemia-causing coronary stenosis by using FFR as the reference standard. CTA and CT-FFR analyses were performed before FFR measurement in a blinded way.

The study was performed in compliance with the Declaration of Helsinki and Good Clinical Practice guidelines of the China Food and Drug Administration. The study protocol was approved by the institutional review board at each site. All patients provided written informed consent.

### Study Subjects

**FIGURE 1:**
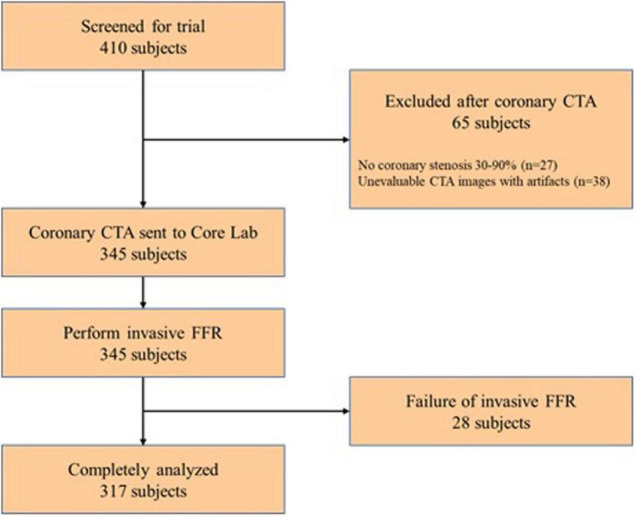
Study flowchart. CTA, computed tomography angiography; FFR, fractional flow reserve.

Adults indicated for invasive coronary angiography (ICA) due to high risk of significant coronary stenosis between November 2018 and March 2020, were prospectively enrolled. Patients were deemed not eligible if they had prior stent implantation or coronary bypass surgery, allergy to the contrast agent, nitrates, or adenosine, suspicion of acute coronary syndrome, serum creatinine > 150 μmol/L, or a glomerular filtration rate < 45 ml/kg/1.73 m^2^, severe heart failure, pregnant state. CTA inclusion criteria were as follows: ≥1 stenosis with percent diameter stenosis between 30 and 90% in a vessel with diameter ≥ 2.0 mm. CTA exclusion criteria were as follows: significant arrhythmia, poor image quality, Agatston score ≥ 800. Angiographic exclusion criteria were: the interrogated stenosis is caused by myocardial bridge; ostial lesions less than 3 mm to the aorta; poor angiographic image quality precluding contour detection; severe overlap of stenotic segments, and severe tortuosity of target vessel. The flowchart of this study is displayed in Figure 1.

### Coronary Computed Tomography Angiography

Coronary CTA was performed with CT scanner available at each participating hospital (Revolution CT, GE Healthcare, Milwaukee, WI, United States; SOMATOM Definition Flash/Force, Siemens Healthineers, Forchheim, Germany). Oral beta-blockers were used to achieve target heart rate <80 beats/min. Sublingual nitrates were given immediately before image acquisition. Scanning parameters were set as follows: tube voltage 100 kV (<60 kg) or 120 kV (>60 kg), detector collimation, 256 × 0.625/64 × 2 × 0.6/96 × 2 × 0.6 mm; gantry rotation time, 280/280/250 ms. Image acquisition was prospectively triggered to the patient’s electrocardiogram at 35–75% of the R-R interval, with a section thickness of 0.625/0.75/0.75 mm, a reconstruction increment of 0.625/0.5/0.5 mm and a reconstructed field of view of 200–250 mm. Contrast medium (Ultravist 370 mgI/ml, Bayers-Schering Pharma, Berlin, Germany) of 50–60 ml was injected through an antecubital vein using a double-head power injector (Stellant, Medrad, Pittsburgh, PA, United States) with an injection rate of 4.5–5.5 ml/s. A triple phase contrast protocol was used as follows: pure contrast material in phase I, contrast medium/saline mixture by 30/70% in phase II, and pure saline in phase III.

### Computational Fluid Dynamics Based Computed Tomography-Fractional Flow Reserve Computation

Computational fluid dynamics modeling of CT-FFR calculation software system was developed by Heartcentury co., Ltd. (Beijing, China). This new parameter-optimized CFD algorithm was developed by two major steps. The first is the anatomic model construction. Aortic root and left ventricle myocardium were segmented using a customized 3D semantic segmentation by deep learning. Coronary centerlines were traced from ostia to distal vessels through vessel enhancement. Coronary lumen boundaries were detected by contour delineation in radial directions perpendicular to centerlines. Anatomic models were constructed by merging the geometric models of coronary arteries and the aortic root. The next step is CT-FFR computation. The parameters used in the boundary conditions for CT-FFR computation were optimized in reduced-order models using retrospective data by minimizing the difference between CT-FFR and known FFR. The parameters were fixed and applied to all subjects during this prospective trial. Using meshed anatomic models constructed from CTA and boundary conditions with optimized parameters, computation based on a customized CFD solver was performed until sufficient convergence to determine velocity and pressure at each vertex under steady flow conditions. Blood was assumed to be incompressible Newtonian fluid with a density of 1.06 g/cm^3^ and a dynamic viscosity of 0.04 g/cm⋅s. Finally, CT-FFR was calculated as the ratio of the pressure of any vertex in the mesh to the pressure in the aorta at the coronary ostium.

### Quantitative Analysis of Coronary Atherosclerosis Burden

Coronary plaque burden was performed with a dedicated coronary plaque analysis software (Syngo.via vb10b, Siemens) in a core laboratory. The software automatically calculated lesion length (calculated as the centerline distance from the proximal to distal end of the target lesion), lumen diameter and lumen area of stenosis, plaque burden (calculated as plaque volume divided by total vessel volume), total plaque volume, non-calcified plaque volume and calcified plaque volume both on target vessel level and target lesion level.

### Coronary Angiography and Fractional Flow Reserve Measurement

Angiographic images were recorded at 15 frames/s by monoplane or biplane radiographic systems (AXIOM Artis, Siemens Healthcare, Erlangen, Germany; Innova, GE, Wauwatosa, WI, United States). Catheterization was performed by means of either femoral or radial route. Recommended standard projection views were obtained for angiographic image acquisition.

After routine coronary angiography, the operators measured FFR using RadiAnalyzer Xpress instrument and Certus pressure wire (Abbott Vascular, Santa Clara, CA, United States). Hyperemia was induced by intravenous administration of adenosine-triphosphate (ATP) *via* the antecubital vein at a rate of 140–180 μg/kg per minute. Pressure data were recorded for at least 3 s of stable value before ATP administration and at least 10 s of stable value during hyperemia. The pressure sensor was returned to the guiding catheter tip to exclude pressure drift. FFR was measured 2 to 3 cm distal to the stenosis. FFR < 0.80 was defined as the presence of lesion-specific ischemia, while the range between 0.75 and 0.8 was considered as the “gray zone.”

### Core Laboratory Analysis

All CTA images, coronary angiography data and invasive FFR measurements were sent to an independent core laboratory for analysis. CTA, ICA, CT-FFR calculation, and FFR reading were analyzed independently in a blinded way. One observer (NZ, with 3 years of experience in coronary CTA), blinded to the results of invasive FFR, measured CT-FFR 2 to 3 cm distal to the stenosis for each target vessel.

### Statistical Analysis

Quantitative variables were expressed as mean ± SD if normally distributed, and compared using 2-sample Student *t*-tests for independent samples. Median and interquartile range (IQR) were provided for skewed data and compared using non-parametric Mann-Whitney *U* tests. Categorical variables were presented as absolute values, and percentages were compared between groups using chi-square tests or Fisher’s exact tests as needed. Sensitivity, specificity, accuracy, positive predictive value, and negative predictive values with their corresponding 95% confidence intervals (CIs) were calculated for CTA and CT-FFR, respectively. Bland–Altman plot was performed with MedCalc version 18 (MedCalc Software, Mariakerke, Belgium) and other statistical analyses were performed with SPSS software package (version 18.0, IBM Corp., Armonk, NY, United States). The univariate logistic regression model was used to evaluate the effects of characteristics on false negative and false positive CT-FFR in vessels with different FFR categories. A *p*-value < 0.05 was considered statistically significant.

## Results

### Baseline Patient and Lesion Characteristics

Among 410 subjects undergoing study screening between September 2018 and March 2020, a total of 65 subjects were excluded after coronary CTA, and 28 subjects were excluded due to failure of measuring FFR. A total of 317 patients (68.5% men, mean age 59.4 ± 9.7 years) were available for the primary endpoint analysis (Figure 1). Comparison of invasive FFR and CT-FFR was performed on 366 vessels. Baseline characteristics of patients and coronary CTA were provided in Table 1. The mean Agatston score was 246.6. Only 1 patient had serious adverse event of coronary dissection during percutaneous coronary intervention (PCI) treatment after FFR measurement.

**TABLE 1:** Demographic and clinical characteristics of participants.

Characteristics	*N*
Male	217 (68.5)
Age (years)*	59.4 ± 9.7
BMI, kg/m^2^	25.7 ± 3.8
Hypertension	196 (61.8)
Diabetes mellitus	98 (30.9)
Hyperlipidemia	201 (65.3)
Smoking	153 (48.3)
Family history of CAD	41 (12.9)
History of Stroke	25 (7.9)
Peripheral vascular disease	10 (3.2)
**Chest pain**	
Typical angina	61 (19.2)
Atypical angina	142 (44.8)
Non-angina chest pain	114 (36.0)
Heart rate during CTA*	70.5 ± 41.7
Pre-scan administration of beta-blockers	35 (11.1)

*Except where indicated, data are numbers of patients, with percentages in parentheses.*

* Values are mean ± SD or n (%).

*CAD, coronary artery disease; CTA, computed tomography angiography.*

Fractional flow reserve indicated that hemodynamic stenosis presented in 169 vessels of 162 patients, involving 139 (82.2%) lesions located in LAD, 15 (8.9%) in LCX and 15 (8.9%) in RCA. Per-patient and per-vessel characteristics of coronary CTA, CT-FFR, ICA, and FFR were presented in Table 2.

**TABLE 2:** Characteristics of coronary CTA, CT-FFR, ICA, and FFR on per-patient and per-vessel level.

Characteristics	*N*
Patient level	317
Agatston score	88.5 (16.8, 331.2)
Patients with agatston score 100–400	96 (30.5)
Patients with agatston score > 400	24 (7.6)
Patients with coronary CTA maximum stenosis ≥ 50%	253 (79.8)
Patients with coronary CTA maximum stenosis ≥ 70%	133 (42.0)
Patients with CT-FFR < 0.8	149 (47.0)
Patients with ICA maximum stenosis ≥ 50%	206 (65.0)
Patients with ICA maximum stenosis ≥ 70%	76 (24.0)
Patients with FFR < 0.8	161 (50.8)
Vessel level	366
Vessels with coronary CTA maximum stenosis ≥ 50%	278 (76.0)
Vessels with coronary CTA maximum stenosis ≥ 70%	140 (38.3)
Vessels with CT-FFR < 0.8	174 (47.5)
Vessels with ICA maximum stenosis ≥ 50%	229 (62.6)
Vessels with ICA maximum stenosis ≥ 70%	82 (22.4)
Vessels with FFR < 0.8	169 (46.2)
Vessels with FFR 0.76–0.8	50 (13.7)

*Except where indicated, data are numbers of patients, with percentages in parentheses.*

*CTA, computed tomography angiography; ICA, invasive coronary angiography; FFR, fractional flow reserve; CT-FFR, fractional flow reserve derived from computed tomography.*

### Correlation and Agreement Between Computed Tomography-Fractional Flow Reserve and Fractional Flow Reserve

**FIGURE 2:**
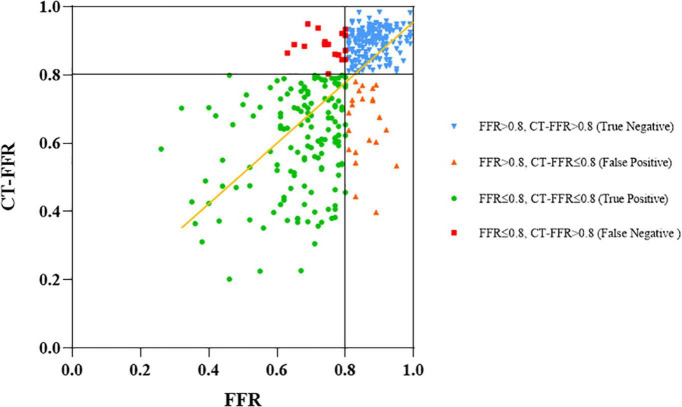
Correlation between CT-FFR and invasive FFR. Measurements of CT-FFR and FFR are shown as a scatter plot (black lines show the 0.8 cutoffs and yellow line shows the fitted line).

**FIGURE 3:**
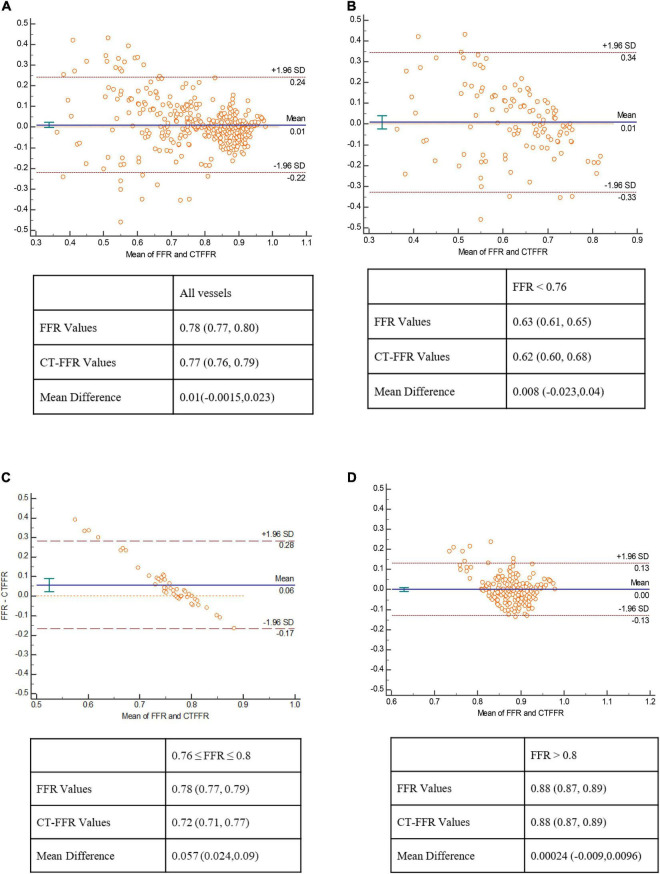
Bland–Altman plot of CT-FFR and FFR. Bland–Altman analysis show a very mild systematic underestimation of CT-FFR with FFR values in all lesions **(A)**, and in lesions with FFR < 0.76 **(B)**, a relatively large underestimation of CT-FFR with FFR in gray zone lesions **(C)**, and a nearly equal value of CT-FFR and FFR in non-ischemia lesions **(D)**.

There was good correlation between CT-FFR and FFR values on per-vessel level [Pearson’s correlation coefficient 0.72 (*p* < 0.001)]. The scatter plot was shown in Figure 2. The mean values of CT-FFR and FFR were quite similar except the lesions located in gray zone (0.72 vs. 0.78). Bland–Altman analysis shown in Figure 3 demonstrated a slight underestimation of CT-FFR compared with FFR for all vessels (mean difference 0.01, 95% limits of agreement: –0.0015, 0.023), a very slight underestimation of CT-FFR for non-ischemic vessels (mean difference 0.00024, 95% limits of agreement: –0.009, 0.0096), and a higher underestimation of CT-FFR in gray zone vessels (mean difference 0.057, 95% limits of agreement: 0.024, 0.09).

### Diagnostic Performance of Computed Tomography-Fractional Flow Reserve and Computed Tomography Angiography

**FIGURE 4:**
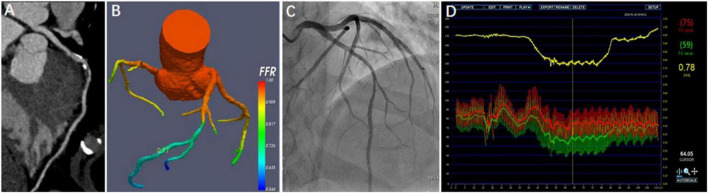
Representative case from the study. A 45-year-old man with atypical angina. CTA showed a 50–70% stenosis in the middle segment of the left anterior descending artery (LAD) **(A)**, and CT-FFR value (0.77) was measured in the distal of target lesion **(B)**. Invasive coronary angiography (ICA) demonstrated the severe stenosis of 70% **(C)**. FFR measured at the corresponding location was 0.78 **(D)**.

Diagnostic sensitivity, specificity, accuracy, PPV, and NPV for CT-FFR and CTA on a per-patient level were 90.7, 90.4, 90.5, 90.7, 90.4 and 88.9, 37.2, 63.4, 59.3, 76.3%, respectively. On a per-vessel level, the values were 89.9, 87.8, 88.8, 87.3, 91.1 and 89.3, 35.5, 60.4, 54.3, 79.5%, respectively. The diagnostic performance was listed in Table 3. Per-patient and per-vessel area under curve (AUC) of CT-FFR and CTA were 0.92 (95% CI: 0.88, 0.95), 0.89 (95% CI: 0.86, 0.92), and 0.64 (95% CI: 0.58, 0.69), 0.66 (95% CI: 0.60, 0.70), respectively. A case was represented in Figure 4.

**TABLE 3:** Per-patient and per-vessel diagnostic performance of coronary CTA and CT-FFR.

	Per-patient			Per-vessel		
	CTA stenosis ≥ 50%	CT-FFR ≤ 0.8	*P* Value	CTA stenosis ≥ 50%	CT-FFR ≤ 0.8	*P* Value
Sensitivity (%)	88.9 (143/161) [85.1, 94.2]	90.7 (146/161) [86.4, 95.2]	0.474	89.3 (151/169) [84.3, 93.2]	89.9 (152/169) [84.1, 94.3]	1.000
Specificity (%)	37.2 (58/156) [25.5, 39.2]	90.4 (141/155) [90.1, 98.3]	<0.0001	35.5 (70/197) [29.4, 43.3]	87.8 (173/197) [80.1, 90.5]	<0.0001
Accuracy (%)	63.4 (201/317) [56.2, 67.3]	90.5 (288/317) [87.7, 94.2]	<0.0001	60.4 (221/366) [55.5, 65.1]	88.8 (325/366) [85.4, 92.6]	<0.0001
PPV (%)	59.3 (143/241) [52.3, 64.5]	90.7 (146/161) [84.2, 94.7]	<0.0001	54.3 (151/278) [48.3, 60.1]	87.3 (152/174) [81.1, 91.6]	<0.0001
NPV (%)	76.3 (58/76) [65.2, 85.4]	90.4 (141/156) [85.3, 95.5]	0.04	79.5 (70/88) [70.2, 87.6]	91.1 (173/190) [89.4, 96.2]	0.006
AUC	0.64 [0.58, 0.69]	0.92 [0.88, 0.95]	<0.0001	0.66 [0.60, 0.70]	0.89 [0.86, 0.92]	<0.0001

*Numerators and denominators are in parentheses; 95% confidence intervals are in brackets.*

*NPV, negative predictive value; PPV, positive predictive value; AUC, area under the curve; CTA, computed tomographic angiography; CT-FFR, fractional flow reserve derived from computed tomography.*

### Diagnostic Accuracy of Computed Tomography-Fractional Flow Reserve in Ischemia, Gray Zone, and Non-ischemia Vessels

**FIGURE 5:**
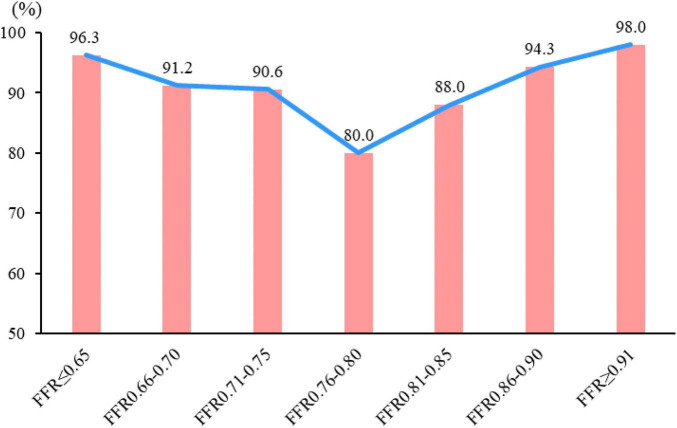
Diagnostic accuracy of CT-FFR in different FFR categories. The diagnostic accuracy of CT-FFR among the different FFR groups in correctly identifying the hemodynamically ischemic lesions is shown. The diagnostic accuracy of lesions in “gray zone” lesions (FFR = 0.75–0.80) was the lowest, and the farther from the gray zone, the higher the accuracy.

On a per-vessel basis, the diagnostic accuracy of CT-FFR (≤0.8 or >0.8) was 96.3, 91.2, 90.6, 80, 88, 94.3, and 98% in vessels with FFR < 0.66, 0.66–0.7, 0.71–0.75, 0.76–0.8, 0.81–0.85, 0.86–0.9, and >0.9, respectively. The diagnostic accuracy was relatively lower in gray zone lesions (Figure 5).

### Characteristics of Atherosclerosis Burden in Gray Zone Lesions

When compared between vessels with FFR < 0.76 and gray zone, only lumen area and diameter of target lesion were significantly larger in gray zone lesions (*p* < 0.001). Other plaque burden parameters had no significant differences. On the contrary, when compared between vessels with gray zone and FFR > 0.8, plaque burden parameters including total plaque burden, non-calcified plaque volume of target vessel, lesion length, plaque volume, non-calcified volume and calcified volume of target lesion were all significantly higher in gray zone vessels (all *p* < 0.05) except for lumen area and diameter of target lesion (Table 4).

**TABLE 4:** Coronary atherosclerosis burden of lesions in “gray zone.”

	FFR ≤ 0.75 vs. FFR 0.76–0.8			FFR 0.76–0.8 vs. FFR > 0.8		
	FFR ≤ 0.75	FFR 0.76–0.8	*P* Value*	FFR 0.76–0.8	FFR > 0.8	*P* Value^†^
Total plaque burden of target vessel (%)	34.6 (30.8, 39.0)	33.2 (29.1, 38.6)	0.381	33.2 (29.1, 38.6)	30.2 (26.4, 36.5)	0.003
Non-calcified plaque volume of target vessel (mm^3^)	60.5 (26.4, 114.8)	58.4 (26.1, 111.1)	1.000	58.4 (26.1, 111.1)	34.3 (15.2, 71.0)	0.024
Lesion length (mm)	22.3 (14.0, 34.9)	21.3 (15.6, 33.6)	1.000	21.3 (15.6, 33.6)	17.6 (11.0, 26.0)	0.012
Plaque volume of target lesion (mm^3^)	153 (65.2, 254.8)	159.5 (113.3,241.2)	1.000	159.5 (113.3,241.2)	87 (39.3, 180.9)	0.000
Non-calcified plaque volume of target lesion (mm^3^)	20.9 (9.2, 44.7)	18.2 (9.6,42.3)	1.000	18.2 (9.6,42.3)	12.5 (4.6, 26.9)	0.007
Calcified plaque volume of target lesion (mm^3^)	11.7 (0.72, 52.8)	21.6 (3.4,72.7)	0.290	21.6 (3.4,72.7)	7.6 (0.1, 29.9)	0.008
Lumen area of target lesion (mm^2^)	1.81 (1.17, 2.38)	2.3 (1.65,3.05)	0.012	2.3 (1.65,3.05)	2.5 (1.86, 3.12)	0.500
Lumen diameter of target lesion (mm)	1.52 (1.22, 1.74)	1.71 (1.46,1.97)	0.011	1.71 (1.46,1.97)	1.78 (1.54, 2)	0.516

*Data are median, interquartile range are in parentheses.*

*Comparison of coronary plaque burden between lesions with FFR ≤ 0.75 and 0.76–0.8. ^†^Comparison of coronary plaque burden between lesions with FFR > 0.8 and 0.76–0.8. FFR, fractional flow reserve.

### False Positive and False Negative Analysis of Computed Tomography-Fractional Flow Reserve

Table 5 listed the CTA characteristics on false positive (FP) and false negative (FN) lesions by CT-FFR in different FFR level. In lesions with FFR < 0.76, 7 of the 119 lesions (5.9%) were misdiagnosed by CT-FFR (CT-FFR > 0.8). When comparing false negative (FN) lesions with true positive (TP) lesions, lumen area and diameter of target lesion were both significantly larger in FN lesions (both *p* < 0.05). In gray zone (FFR, 0.76–0.80) lesions, 10 of the 50 lesions (20%) were misdiagnosed by CT-FFR (CT-FFR > 0.8). Coronary artery calcium score (CACS) and calcified plaque volume of target lesion were significantly lower in false negative lesions (*p* = 0.037 and 0.034, respectively). In lesions with FFR > 0.8, 21 of the 197 lesions (10.7%) were misdiagnosed by CT-FFR (CT-FFR ≤ 0.8). When comparing false positive (FP) lesions with true negative (TN) lesions, CACS, plaque burden and calcified volume of target lesion were significantly higher in false positive lesions (*p* = 0.029, 0.033, and 0.012, respectively).

**TABLE 5:** Computed tomography angiography characteristics on FP and FN lesions by CT-FFR in different FFR level.

	FFR < 0.76 (*n* = 119)			0.76 ≤ FFR ≤ 0.8 (*n* = 50)			FFR > 0.8 (*n* = 197)		
	CT-FFR > 0.8(FN) (*n* = 7)	CT-FFR ≤ 0.8(TP) (*n* = 112)	*P* Value	CT-FFR > 0.8(FN) (*n* = 10)	CT-FFR ≤ 0.8(TP) (*n* = 40)	*P* Value	CT-FFR > 0.8(TN) (*n* = 176)	CT-FFR ≤ 0.8(FP) (*n* = 21)	*P* Value
LAD	6	87	1.000	8	35	0.616	96	12	0.577
ICA > 50%	6	95	1.000	7	23	0.689	81	13	0.182
Agatston score of target lesion	40.8 (4.8, 126.1)	45.6 (6.1, 194.3)	0.566	4.16 (1.16, 47.68)	28.2 (30.26, 67.53)	0.037	29 (0, 120)	118.3 (31.3, 257.5)	0.029
Plaque burden of target lesion (%)	53.2 (41.0, 60.0)	54.6 (47.1, 61.8)	0.658	55.6 (46.7, 59.9)	50.8 (44.2, 58.0)	0.308	46.1 (36.9, 56.0)	56.0 (46.8, 62.6)	0.033
Calcified volume of target lesion (mm^3^)	7.2 (0.8, 42.7)	9.0 (0.2, 53.1)	0.630	4.5 (0, 51)	38.2 (8.2, 94.2)	0.034	4.2 (0, 28.3)	27.1 (8.5, 83.1)	0.012
Lumen area of target lesion (mm^2^)	2.5 (1.7, 3.6)	1.7 (1.2, 2.4)	0.048	2.6 (2.4, 3.1)	2.2 (1.6, 2.9)	0.119	2.5 (1.9, 3.2)	2.3 (1.7, 2.6)	0.128
Lumen diameter of target lesion (mm)	1.8 (1.5, 2.2)	1.5 (1.2, 1.7)	0.047	1.8 (1.7, 2.0)	1.7 (1.4, 1.9)	0.119	1.8 (1.5, 2.0)	1.7 (1.5, 1.8)	0.803

*Data are median, interquartile range are in parentheses.*

*Comparison of plaque burden between TP and FN in FFR < 0.76 and 0.76–0.8 groups, TN and FP in FFR > 0.8 group are shown.*

*LAD, left anterior descending artery; ICA, invasive coronary angiography; FFR, fractional flow reserve; CT-FFR, fractional flow reserve derived from computed tomography; FN, false negative; FP, false positive; TN, true negative; TP, true positive.*

The effects of coronary characteristics on FN or FP lesions were investigated by univariate logistic regression analysis (Table 6). In lesions in gray zone, CACS of target lesion [odds ratio (OR) = 1.009, *p* = 0.044] has a significant effect on increasing risk of FN results of CT-FFR. In non-ischemia lesions (FFR > 0.8), coronary stenosis > 50% (OR = 2.684, *p* = 0.03) has a significant effect on increasing risk of FP results of CT-FFR, and lumen area (OR = 0.567, *p* = 0.02) and lumen diameter (OR = 0.296, *p* = 0.03) of target lesion has a significant negative effect on the risk of FP results of CT-FFR.

**TABLE 6:** Univariate logistic regression analysis of the effects of CTA characteristics on FP and FN lesions by CT-FFR in different FFR level.

	FFR < 0.76		0.76 ≤ FFR ≤ 0.8		FFR > 0.8	
	OR	*p* Value	OR	*p* Value	OR	*p* Value
LAD	2.059 (0.374–11.336)	0.407	0.722 (0.262–1.986)	0.528	0.839 (0.501–1.406)	0.505
ICA > 50%	1.021 (0.985–1.021)	0.985	1.582 (0.439–5.705)	0.483	2.684 (1.099–6.553)	0.030
CACS of target lesion	1.001 (0.996–1.006)	0.605	1.009 (1.000–1.018)	0.044	1.000 (0.998–1.002)	0.907
Plaque burden of target lesion	1.001 (0.994–1.008)	0.848	1.023 (0.960–1.091)	0.483	1.027 (0.993–1.062)	0.117
Calcified volume of target lesion	1.021 (0.989–1.054)	1.021	1.016 (0.997–1.035)	0.103	1.000 (0.995–1.006)	0.957
Lumen area of target lesion	0.627 (0.383–1.028)	0.064	0.689 (0.416–1.140)	0.147	0.567 (0.3510.915)	0.020
Lumen diameter of target lesion	0.274 (0.06–1.247)	0.094	0.238 (0.047–1.197)	0.082	0.296 (0.098–0.887)	0.030

*Data are median, 95% CI confidence interval are in parentheses.*

*CACS, coronary artery calcium score; FN, false negative; FP, false positive; FFR, fractional flow reserve; CT-FFR, fractional flow reserve derived from computed tomography; CTA, computed tomography angiography; ICA, invasive coronary angiography; LAD, left anterior descending artery.*

## Discussion

The CT-FFR-CHINA trial conducted at multiple centers in China demonstrates superior diagnostic performance of CT-FFR based on a new parameter-optimized CFD algorithm to identify ischemia-causing lesions compared with coronary CTA. This represents the first prospective clinical trial investigating the new algorithm using invasive FFR as the reference standard in China. Importantly, the diagnostic accuracy of “gray zone” lesions is improved.

Up to date, several techniques to compute FFR non-invasively from coronary CTA were validated both in prospective clinical trials and retrospective cohort studies. The NXT trial enrolled 254 subjects, validated against FFR, and reported a per-patient sensitivity of 0.86 and a specificity of 0.79 (15). We demonstrated a higher per-patient sensitivity of 0.91 and a specificity of 0.90 in 317 subjects. Such improvement may be the due to the incremental benefit of the updated CT-FFR algorithm. For cohort studies, the data are analyzed retrospectively and not blinded, the results are less convincing. Adriaan et al. performed CT-FFR in 203 vessels, and reported the sensitivity, specificity and accuracy of CT-FFR were 0.88, 0.65, and 0.75, respectively (18). Brian S. Ko performed 42 patients with 320-detector coronary CTA and found the sensitivity and specificity of CT-FFR was 0.67 and 0.91. While, the trial reported in this study demonstrated a higher per-vessel sensitivity of 0.90 and a specificity of 0.88 in 366 subjects (19). The reported CT-FFR algorithms leverage either CFD, or machine learning (12, 20) applied to geometric models derived from coronary CTA. The difference lies in whether physics-based or learning-based principles are used to establish the relationship between and vascular anatomy in coronary arteries. The accuracy of FFR calculation largely depend on the reliable specification of boundary conditions, which cannot be measured directly from CTA data alone. Therefore, the main novelty of our CFD-based technique is the explicit tuning of the parameters used in the boundary conditions. In the reduced order model for blood pressure and flow in coronary arteries, we showed the computed FFR can be expressed as a function of these parameters. Those parameters were optimized by minimizing the mean squared error of the computed and measured FFRs over all vessels in retrospectively collected data. The optimized parameters then were used in coronary blood flow simulation in full-3D models using our customized CFD solver. Our approach reduces uncertainty in selection of these parameters, which are unseen in the CTA data and required to set under hyperemic conditions. It is also more adaptive better to a targeted population and warrants continuous improvement with the increased number of measurements accumulated over time. Other improvements of our CFD-based technique include adoption of machine/deep learning in geometric construction of vascular anatomy, adaptive meshing in coronary arteries and aorta, and simulation under steady flow conditions. The end to end time to obtain CT-FFR calculation using our technique is 20 min on average, which is acceptable in clinical settings. Compared to previous studies, workstation-based algorithms allow CT-FFR determination in approximately 45 min or less (21, 22), 15–30 min with machine learning-based CT-FFR (19).

Lesions located in “Gray zone” have always been a challenge for CT-FFR. The diagnostic performance in this area inevitably decreases (17, 23). As there was no clear definition of the gray zone in the current guidelines, proposed value between 0.76 and 0.8 was adopted (24). In our study, we also observed a decrease in the performance of CT-FFR for detecting lesion-specific ischemia, and the difference between CT-FFR and FFR values was also the largest. The mean value of CT-FFR was 0.72 in gray zone, indicating that most lesion were overestimated by CT-FFR. Previous study found that plaque markers including lesion length, non-calcified plaque volume and napkin-ring sign were portending predictive value to identify lesion-specific ischemia (25, 26). Further analysis of our data revealed that less severe lumen stenosis in gray zone was mainly different from the real ischemia vessels (FFR < 0.76), and the obviously increased plaque burden in gray zone was mainly different from the non-ischemia vessels.

From the further analysis of CT-FFR misdiagnosed lesions, we found that false negative rate of CT-FFR was 5.9% in lesions with FFR < 0.76, Whereas the rate increased to 20% for lesions in gray zone. For lesions in gray zone, calcified plaque had a significant effect on increasing risk of false negative results of CT-FFR. False positive rate of CT-FFR was 10.7% in non-ischemia lesions (FFR > 0.8), and the degree of coronary stenosis had a significant effect on increasing risk of false positive results of CT-FFR. Therefore, it was important to continue to improve the diagnostic accuracy of CT-FFR in calcified plaque, and the diagnostic capabilities of CT-FFR will be evaluated through further studies with larger sample sizes.

## Study Limitations

This current clinical trial had some inherent limitations. Firstly, CT-FFR was performed by experienced operators in the core lab for reproducibility and stability. Thus, there may be some inconsistency in the results when analyzed in local labs. Therefore, rigorous training is needed for the operators. Secondly, the effect of significant calcification on CT-FFR could not be adequately analyzed because there were only 24 patients with Agatston score > 400. As calcified plaque is the major factor affecting the diagnostic accuracy of CT-FFR, it is essential to enroll more patients with multiple calcified plaque and make a further analyzation in the future.

## Conclusion

The CT-FFR-CHINA trial confirmed that the new parameter optimized CFD algorithm-based CT-FFR provides higher diagnostic performance, In particular the specificity for lesion-specific ischemia, than CTA in Chinese population, and may have an acceptable accuracy of gray zone lesions. The stenosis degree in gray zone was less than lesions with FFR < 0.76, while the plaque load was heavier than non-ischemic lesions.

## Data Availability Statement

The original contributions presented in the study are included in the article, further inquiries can be directed to the corresponding author.

## Ethics Statement

The studies involving human participants were reviewed and approved by IRB of all sites participating in the study. The patients/participants provided their written informed consent to participate in this study.

## Author Contributions

LS, HH, TJ, WC, FZ, KD, CM, WY, GF, LX, DL, LF, and BX managed the clinical trial. YG, NZ, and YA performed the material preparation and data collection. YW and WL performed data analysis. YG completed the draft of the manuscript. All authors read the study and approved the manuscript for publication.

## Conflict of Interest

The authors declare that the research was conducted in the absence of any commercial or financial relationships that could be construed as a potential conflict of interest.

## Publisher’s Note

All claims expressed in this article are solely those of the authors and do not necessarily represent those of their affiliated organizations, or those of the publisher, the editors and the reviewers. Any product that may be evaluated in this article, or claim that may be made by its manufacturer, is not guaranteed or endorsed by the publisher.
